# Immunoreactive Proteins of *Bifidobacterium longum* ssp. *longum* CCM 7952 and *Bifidobacterium longum* ssp. *longum* CCDM 372 Identified by Gnotobiotic Mono-Colonized Mice Sera, Immune Rabbit Sera and Non-immune Human Sera

**DOI:** 10.3389/fmicb.2016.01537

**Published:** 2016-09-29

**Authors:** Sabina Górska, Ewa Dylus, Angelika Rudawska, Ewa Brzozowska, Dagmar Srutkova, Martin Schwarzer, Agnieszka Razim, Hana Kozakova, Andrzej Gamian

**Affiliations:** ^1^Department of Medical Microbiology, Ludwik Hirszfeld Institute of Immunology and Experimental Therapy of the Polish Academy of SciencesWroclaw, Poland; ^2^Laboratory of Gnotobiology, Institute of Microbiology, Academy of Sciences of the Czech Republic v. v. i.,Novy Hradek, Czech Republic

**Keywords:** *Bifidobacterium*, probiotics, moonlighting proteins, immunoreactivity, surface proteins

## Abstract

The *Bifidobacteria* show great diversity in the cell surface architecture which may influence the physicochemical properties of the bacterial cell and strain specific properties. The immunomodulatory role of bifidobacteria has been extensively studied, however studies on the immunoreactivity of their protein molecules are very limited. Here, we compared six different methods of protein isolation and purification and we report identification of immunogenic and immunoreactive protein of two human *Bifidobacterium longum* ssp. *longum* strains. We evaluated potential immunoreactive properties of proteins employing polyclonal sera obtained from germ free mouse, rabbit and human. The protein yield was isolation method-dependent and the reactivity of proteins detected by SDS-PAGE and Western blotting was heterogeneous and varied between different serum samples. The proteins with the highest immunoreactivity were isolated, purified and have them sequenced. Among the immunoreactive proteins we identified enolase, aspartokinase, pyruvate kinase, DnaK (*B. longum* ssp. *longum* CCM 7952) and sugar ABC transporter ATP-binding protein, phosphoglycerate kinase, peptidoglycan synthethase penicillin-binding protein 3, transaldolase, ribosomal proteins and glyceraldehyde 3-phosphate dehydrogenase (*B. longum* ssp. *longum* CCDM 372).

## Introduction

Bifidobacteria are nonpathogenic, anaerobic, non-motile, non-sporulating, non-gas producing, branched rod-shaped Gram-positive bacteria highly represented in the normal microbiota of the human and animals gastrointestinal tract. They were first isolated from feces of a breast-fed infant by Tissier in 1899, and then named *Bacillus bifidus* (Tisseir, 1900). The *Bifidobacterium* genus comprises 48 species, exhibits a wide spectrum of metabolic properties and possess genomes of a high G+C content (60%) (Ventura et al., 2007; Endo et al., 2012; Killer et al., 2009, 2011). They are widely used in food and pharmaceutical industry as a synbiotic or probiotics due to their health-promoting effects on the human host (Gaggia et al., 2011). Certain Bifidobacteria exert a range of health benefits, including the regulation of intestinal microbial homeostasis, the inhibition of pathogens and harmful bacteria that colonize and/or infect the gut mucosa, the modulation of local and systemic immune responses, the repression of procarcinogenic enzymatic activities within the microbiota, the production of vitamins, and the bioconversion of a number of dietary compounds into bioactive molecules (Singh et al., 1997; Picard et al., 2005). It has been already accepted that probiotic effects of *Bifidobacterium* strain is strictly strain dependent and should not be assigned to other strains, even from the same species. For example, *B. longum* BB536 is claimed to alleviating symptoms of allergy by promoting Th1 response, whereas *B*. *longum* ACTT15707 showed a strong induction of interleukin 10 (IL-10) and could help in the treatment and prevention of gastrointestinal infection (Sanz et al., 2007). Recently, Srutkova et al. (2015) have shown that *B*. *longum* ssp. *longum* CCM 7952, but not *B*. *longum* ssp. *longum* CCDM 372, protected mice from the development of experimental colitis suggesting that careful selection might be crucial in providing beneficial outcome in clinical trials with probiotics bacteria.

Our understanding of bifidobacteria-host interactions, their symbiosis and how bacteria are able to differentially modulate the host immune response is growing, but it is still in its infancy. Little is known about the molecular mechanism of this relationship and the specific bacterial components responsible for the beneficial effect are generally unrecognized. The immune effects of probiotics can be exerted directly by live microbial cells, but also by bacterial cell components localized either in the cell wall or membrane fraction or secreted compounds.

*Bifidobacterium* produces plenty of cell-associated molecules which may play a key role in host-microbiota interactions i.e., polysaccharide antigens which may contribute in bacterial adherence to the host cells (Salazar et al., 2009; Fanning et al., 2012), pili-like structures mediate bacterial colonization (Turroni et al., 2013) or a serpin-like protease inhibitor taking part in host-microbe interaction in the gut (Turroni et al., 2010). Extracellular proteins produced by bifidobacteria are known to be either released into environment or surface-attached. They may be responsible for the enhancement of the mucosal barrier and immunomodulation due to the possibility of direct interaction with host epithelial or immune cells (Dylus et al., 2013). Some other proteins, such as cytosolic proteins: metabolic enzymes, housekeeping, ribosomal proteins and molecular chaperones known as moonlight protein can be also found to be involved in immune cells stimulation, interactions with other bacteria or adhesion phenomena (Henderson and Martin, 2014). It has been demonstrated that in addition to compounds released to culture supernatants, which have clearly an immunomodulation effect (Hoarau et al., 2006), also DNA from different probiotic strains led to cytokine induction (Lammers et al., 2003). However, still the studies on the immunoreactivity of the *Bifidobacterium* cell components are scarce.

The aim of this study was to identify immunoreactive proteins of two *Bifidobacterium* strains: *B*. *longum* ssp. *longum* CCM 7952 and CCDM 372 and compare the efficacy of six different methods used for proteins isolation. The immunoreactivity of proteins was tested by SDS-PAGE and Western blotting using sera from mice mono-colonized with studied strains, sera from rabbits immunized against whole bacteria of studied strains and non-immune human sera. As a result, we have found several major immunoreactive proteins recognized by human sera and an immunogenic proteins which elicit antibody response in mice or rabbit immune system. We also indicated that two methods, namely Heilmann method and extraction by LiCl are usefulness to isolate the immunoreactive proteins.

## Materials and methods

### Bacterial strains and culture conditions

*Bifidobacterium longum* ssp. *longum* CCDM 372 and *Bifidobacterium longum* ssp. *longum* CCM 7952 were originally isolated from feces of breast-fed healthy child. Both strains were provided by Culture Collection of Dairy Microorganisms (Milcom, Czech Republic). The isolates were cultivated in MRS medium (Difco) supplemented with 0.05% L-cysteine-hydrochloride (Millipore) at 37°C in anaerobic conditions (GasPak EZ Anaerobe Container System BD, USA) for 48 h. *Lactobacillus paracsei* LOCK 0912 for sera preparation was isolated form feaces of healthy newborn and cultivated in MRS medium (Difco) at 37°C in microaerophilic conditions for 48 h. Cells were harvested by centrifugation (6000 g for 15 min) (Heraeus Contifuge Stratus, Thermo Scientific, Germany) and washed two times in PBS buffer (phosphate buffered saline pH 7,4). Strain *B*. *longum* ssp. *longum* CCM 7952 is characterized by high amount of slime production, therefore, before protein isolation, the culture was centrifuged at 14,500 g several times to get rid of salts and bacterial polysaccharides.

### Serum samples

#### Mouse non-immune and immune sera

Mouse serum was obtained from germ-free mouse (GF) and gnotobiotic mice mono-colonized with *B. longum* ssp. *longum* CCM 7952 or *B. longum* ssp. *longum* CCDM 372 strains. Germ-free BALB/c mice were kept under sterile conditions in Trexler-type plastic isolators, exposed to 12:12-h light-dark cycles and supplied with autoclaved tap water and 50 kGy irradiated sterile pellet diet Altromin 1410 (Altromin, Lage, Germany) *ad libitum*. Fecal samples were weekly controlled for microbial molds and yeast contamination by standard microbiological methodology. Eight-week-old GF mice were colonized with a single dose (2 × 10^8^ CFU) of freshly grown *Bifidobacterium* strains in 200 μl of sterile PBS by intragastric administration. Stability of colonization was checked by plating of feces on MRS agar supplemented with l-cysteine hydrochloride (0.5 g/l) and CFU were counted after anaerobic cultivation for 48 h at 37°C. Animal experiments were approved by the Animal Experimentation Ethics Committee of the Institute of Microbiology of the Academy of Sciences of the Czech Republic and conducted in accordance with the “European Convention for the Protection of Vertebrate Animals used for Experimental and other Scientific Purposes (CETS No.: 123).”

#### Rabbit immune sera

Rabbit sera were obtained from Termond White rabbit immunized with bacterial mass of *B*. *longum* ssp. *longum* CCM 7952 (R-7952), *B.longum* ssp. *longum* CCDM 372 (R-372) or *L. paracsei* LOCK 0912 (R-0912) as described before (Górska et al., 2016). The experiments were approved by the 1st Local Committee for Experiments with the Use of Laboratory Animals, Wroclaw, Poland (number 12/2012).

#### Human sera

Human adults sera were obtained from healthy volunteers from Military Blood Donors Centre in Wroclaw (Poland, pooled *n* = 10), whereas human umbilical cord sera from healthy women (pooled *n* = 10) were obtained from the Obstetric Clinic of the Medical University of Wroclaw and human adults sera from patients with *Clostridium difficile* infection (pooled *n* = 10) were obtained from 4th Millitary Hospital in Wrocław. The use of sera samples was approved by the Medical Ethics Committee of the Medical University of Wroclaw (number KB-882-2012 and KB-631/2015) and was conducted in accordance with the Helsinki Declaration of 1975. Samples were obtained with patients' written informed consent.

### Protein isolation

Six different methods for protein isolation were tested. The *Bifidobacterium* cell pellets (1 g) were suspended in 5 ml of:

- 10 mM EDTA (Sigma-Aldrich; 30 min., 45°C) according to McCoubrey and Poxton (2001),
- 0,2 M glycine (Sigma-Aldrich) pH 2,2 (30 min, RT) according to Wright et al. (2005),
- 1 M LiCl (Sigma-Aldrich; 30 min, RT) according to McCoy et al. (1975),
- 8 M urea (Sigma-Aldrich; 30 min, RT) according to McCoy et al. (1975),
- 5 M guanidine hydrochloride (Sigma-Aldrich; 2 h, RT) according to Poxton and Byrne (1981),
- 0.5 M Tris-HCl (Serva; pH = 6,8)/SDS (Serva; 0,08% w/v)/glycerol (POCh; 20% v/v)/1 mM β-mercaptoethanol (Sigma-Aldrich) (v/v) and boiled for 5 min according to Heilmann et al. (1996).

After centrifugation (6000 × g for 5 min), proteins were precipitated from the resulting supernatant using 3 volumes of cold 95% ethanol (POCh). Followed by overnight incubation at 4°C, the precipitated proteins were centrifuged (12,000 × g), dissolved in water and dialyzed for 48 h. Protein concentration was measured using the Lowry's method (Lowry et al., 1951). The prepared protein samples were stored in 1 mg aliquots at −70°C.

### SDS-PAGE and Western blot conditions

Equal amounts of proteins samples (10 μg) were analyzed on SDS-PAGE using 5–12.5% gels according to Laemmli (1970) using Tris-Glycine-SDS as running buffer and run for about 2 h at 100 V (Mini-Protean Tetra Cell 165–8001; Bio-Rad, USA). After staining with Coomassie Brilliant Blue (Serva) or soaked in transfer buffer (10 mM Tris-HCl, 150 mM glycine, 20% methanol (POCh) pH 8.3) for 30 min, gels were transferred to a polyvinylidene difluoride membrane (Millipore) for immunoblotting (for 1 h at 100 V). Following transfer, the membrane was blocked with 1% of bovine serum albumin (BSA, KPL), dissolved in phosphate buffered saline, and then incubated with selected sera in 1% BSA for 2 h at RT. We used different dilutions of sera but the most effective was: for rabbit sera 1:5000, mouse sera 1:1000 and for human sera 1:500 (reproducible results, without the Hook effect). After washing in PBS containing 0.25% Tween 20 (PBS-T, Institute of Immunology and Experimental Therapy, PAN) for several times, captured antibodies against protein were detected by incubating the membranes with alkaline phosphatase-labeled goat anti-mouse/rabbit/human IgG antibody (Sigma-Aldrich) diluted 1:10,000. Finally, the membranes were washed as six times with PBS-T, and visualized with solution containing nitro blue tetrazolium (NBT, Roth), 5-bromo-4-chloro-3-indolyl phosphate (BCIP, Roth), and MgCl_2_ (POCh) for 5 s. Image acquisition (exposure time 1–4 min) was performed using VersaDoc Imaging System (Bio-Rad).

### Protein identification

Protein identification was performed as previously described by us (Górska et al., 2016). Briefly, the immunoreactive protein were separated and purified using Prep-Cell apparatus (Model 491 Bio-Rad). Individual spots were cut out from gels and submitted to tryptic digestion, and analyzed by mass spectrometry (spectrometer LC-MS/MS Orbitrap, Thermo). Mascot (Matrix Science, London, UK, http://www.matrixscience.com) and statistical analysis were used to identify proteins from peptide mass fingerprints. All searches were performed against the database for *Bifidobacterium* species. Immunoreactivity of separated proteins was proved using immunoblotting.

## Results

**Figure 1:**
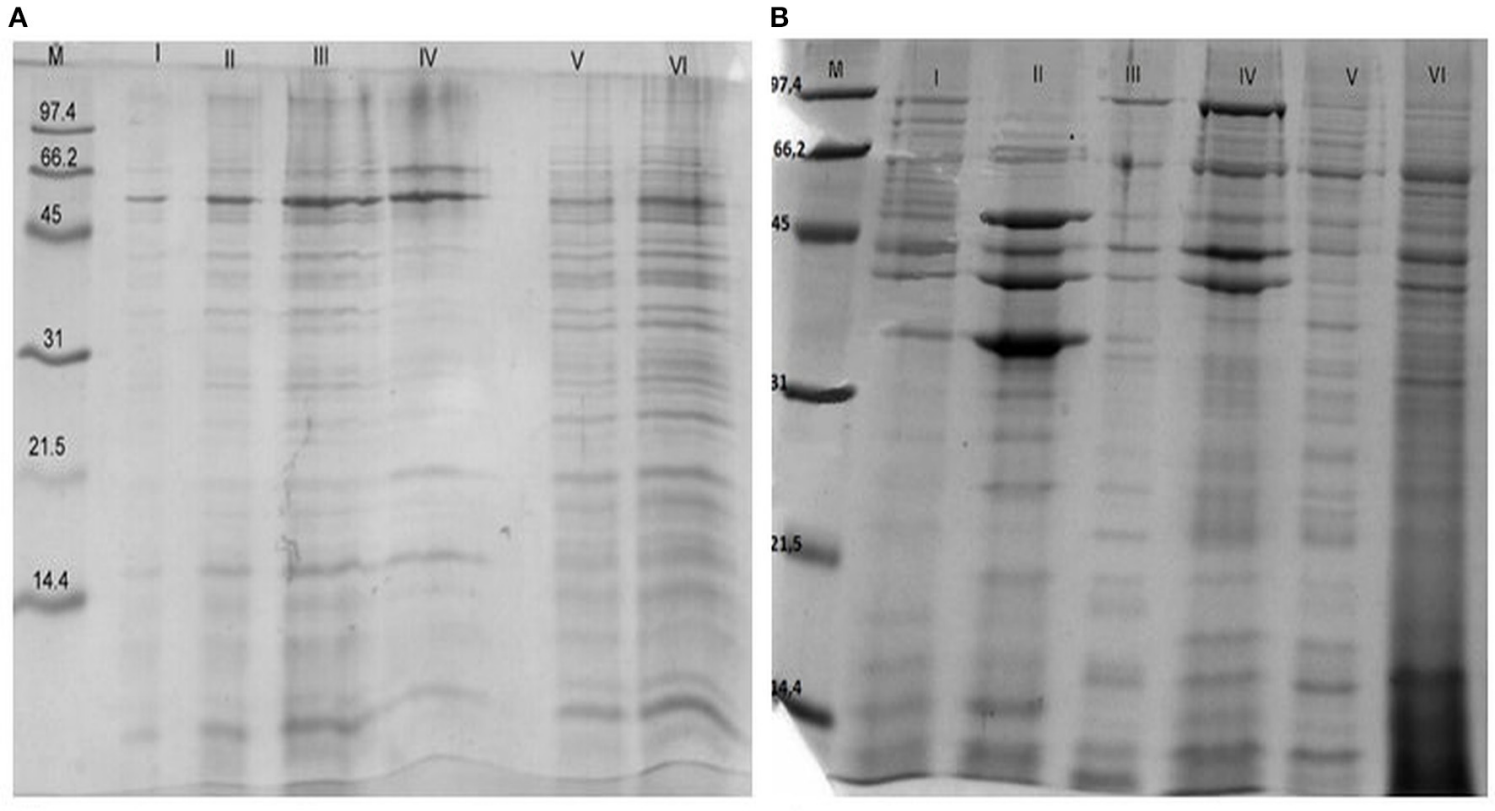
**SDS-PAGE profile of proteins isolated from ***Bifidobacterium longum*** ssp. ***longum*** CCM 7952 (A) and CCDM 372 (B) using different methods: M, molecular mass marker; I, 10 mM EDTA; II, 0,2 M glycine; III, 1 M LiCl; IV, 8 M urea; V, 5 M guanidine hydrochloride; VI, Heilmann methods**. Gels were stained with Coomassie Brilliant Blue.

To investigate the immunoreactive proteins of *B*. *longum* CCM 7952 (CCM 7952) and *B*. *longum* CCDM 372 (CCDM 372) and to identify the differences between these strains we analyzed and compared the profile of reactive proteins obtained with six different methods. The concentration of the protein in extracts varied profoundly and depended on methods and tested strain (Table 1). Generally, we obtained proteins from CCDM 372 with higher yield than from CCM 7952 strain. The protein extracts were run on SDS-PAGE gels and representative results are shown in Figure 1. The profiles varied depending on the isolation method, however we observed several main bands that appeared repeatedly. The samples were immobilized on polyvinylidene difluoride membranes and submitted to immunoblot analysis. The incubation with different immune and non-immune sera revealed the presence of multiple immunoreactive proteins, however only in extracts of four methods, namely: III–1 M LiCl, IV–8 M urea, V–5 M guanidine hydrochloride and VI–Heilmann method. We performed detailed analysis restricted for this four extracts due to the presence of strong immunoreactive bands.

**Table 1:** **The protein concentration in extracts of ***B***. ***longum*** ssp. ***longum*** CCM 7952 and CCDM 372**.

**Method of isolation/*Bifidobacterium* strains**	**10 mM EDTA**	**0,2 M glycine**	**1 M LiCl**	**8 M urea**	**5 M guanidine hydrochloride**	**Heilmann methods**
*B*. *longum* ssp. *longum* CCM 7952	26,3 [μg/ml]	7,1 [μg/ml]	14,0 [μg/ml]	60,9 [μg/ml]	318,6 [μg/ml]	9835 [μg/ml]
*B*. *longum* ssp. *longum* CCDM 372	35,4 [μg/ml]	48,4 [μg/ml]	182,0 [μg/ml]	383,8 [μg/ml]	551,5 [μg/ml]	18238 [μg/ml]

**Figure 2:**
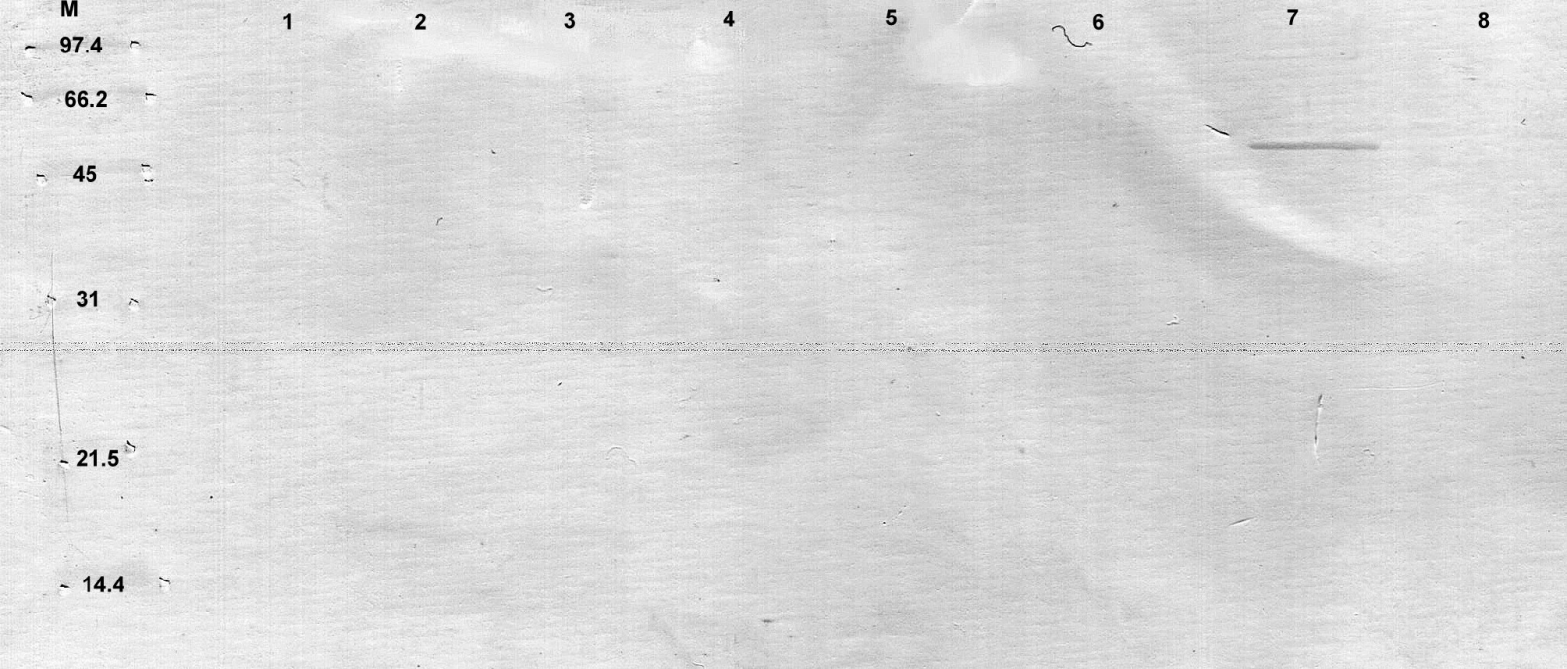
**Immunoblotting of proteins isolated from analyzed strains: M, molecular mass marker [kDa]; line 1, B 372 extract of method III; line 2, B 367 extract of method III; line 3, B 372 extract of method IV; line 4, B 367 extract of method IV; line 5, B 372 extract of method V; line 6, B 367 extract of method V; line 7, B 372 extract of method VI; line 8, B 367 extract of method VI with sera from gnotobiotic mice mon-colonized with ***Bifidobacterium longum*** ssp. ***longum*** CCDM 372**.

In case of mouse sera, we observed that neither protein extracts of CCDM 372 nor CCM 7952 reacted with non-immune sera from germ-free mouse or germ-free mouse mono-colonized with CCM 7952 strain. However, a protein with the molecular weight of about 40 kDa isolated from strain CCDM 372 by Heilmann method (Heilmann et al., 1996) reacted with sera obtained from mice mono-colonized with CCDM 372 strain (Figure 2).

**Figure 3:**
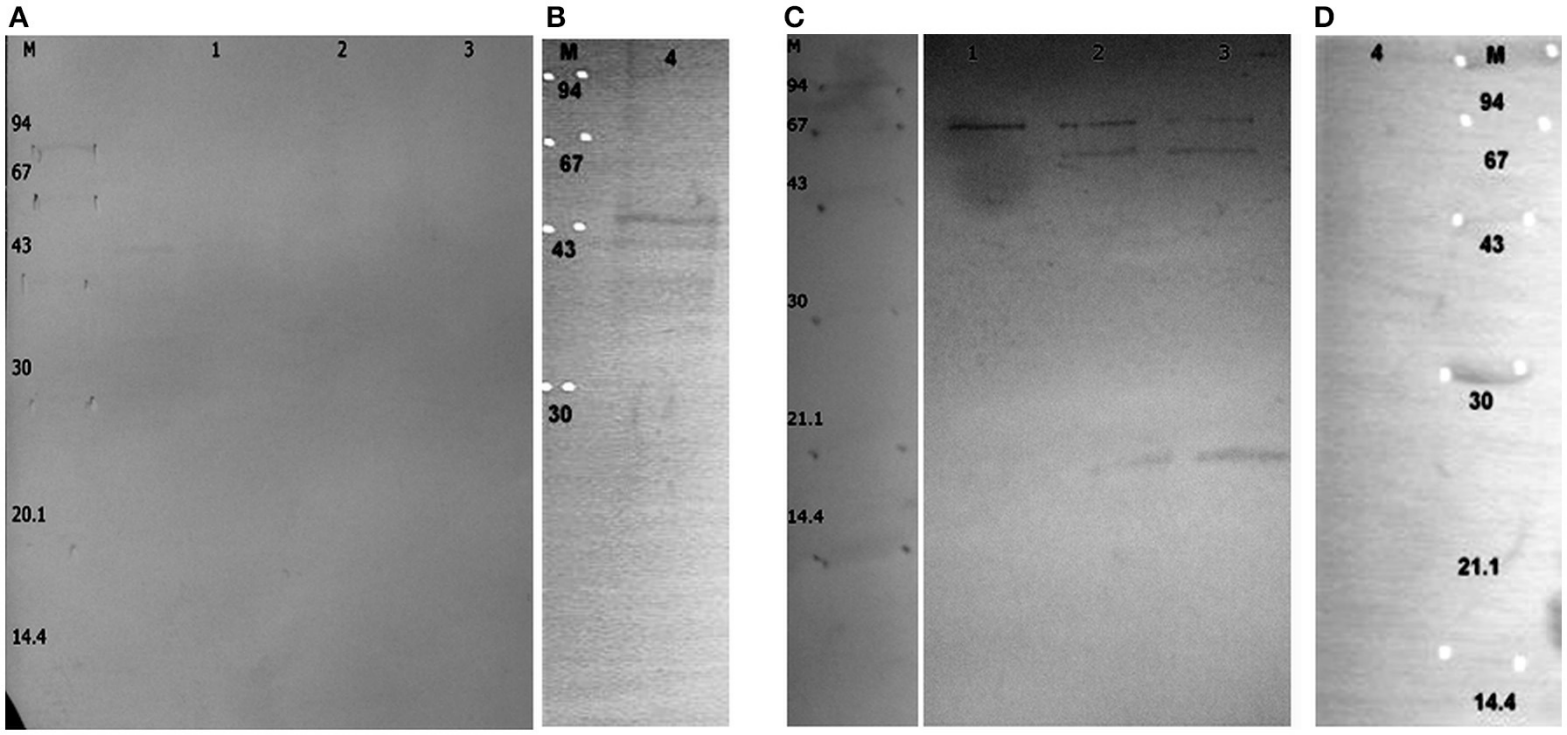
**Immunoblotting of proteins isolated from ***Bifidobacterium longum*** ssp. ***longum*** CCM 7952**. M, molecular mass marker [kDa]; line 1, protein extract of method III; line 2, protein extract of method IV; line 3, protein extract of method V; line 4, protein extracts of method VI; with sera against *Bifidobacterium longum* ssp. *longum* CCM 7952 **(A,B)** and *Bifidobacterium longum* ssp. *longum* CCDM 372 **(C,D)**. These images were taken from different blots, merged and the appropriate scaling was used.

To determine potential immunogenic proteins, we immunized rabbits with whole cells of *B*. *longum* ssp. *longum* CCM 7952 or *B. longum* ssp. *longum* CCDM 372 and obtained polyclonal sera against them: R-7952 and R-372, respectively. We observed that both polyclonal rabbit sera reacted with extract of both strains, however the profiles were quite variable, emphasizing the differences in strains. In case of B. *longum* ssp. *longum* CCM 7952, we detected one strong reaction of protein with molecular weight of about 43 kDa (Figure 3B) reacting with homologous sera R-7952. It must be underline that reactive protein was successfully isolated using Heilmann method (Heilmann et al., 1996) but not by other methods (Figure 3A). However, we observed three strong bands with the molecular weight of about 67 kDa (Figure 3C, line 1, 2, 3), 55 kDa (Figure 3C line 2, 3), 20 kDa (Figure 3C, line 2, 3) cross-reacting with sera against strain *B*. *longum* ssp. *longum* CCDM 372. Interestingly, there was no reaction of protein isolated using Heilmann method (Figure 3D).

**Figure 4:**
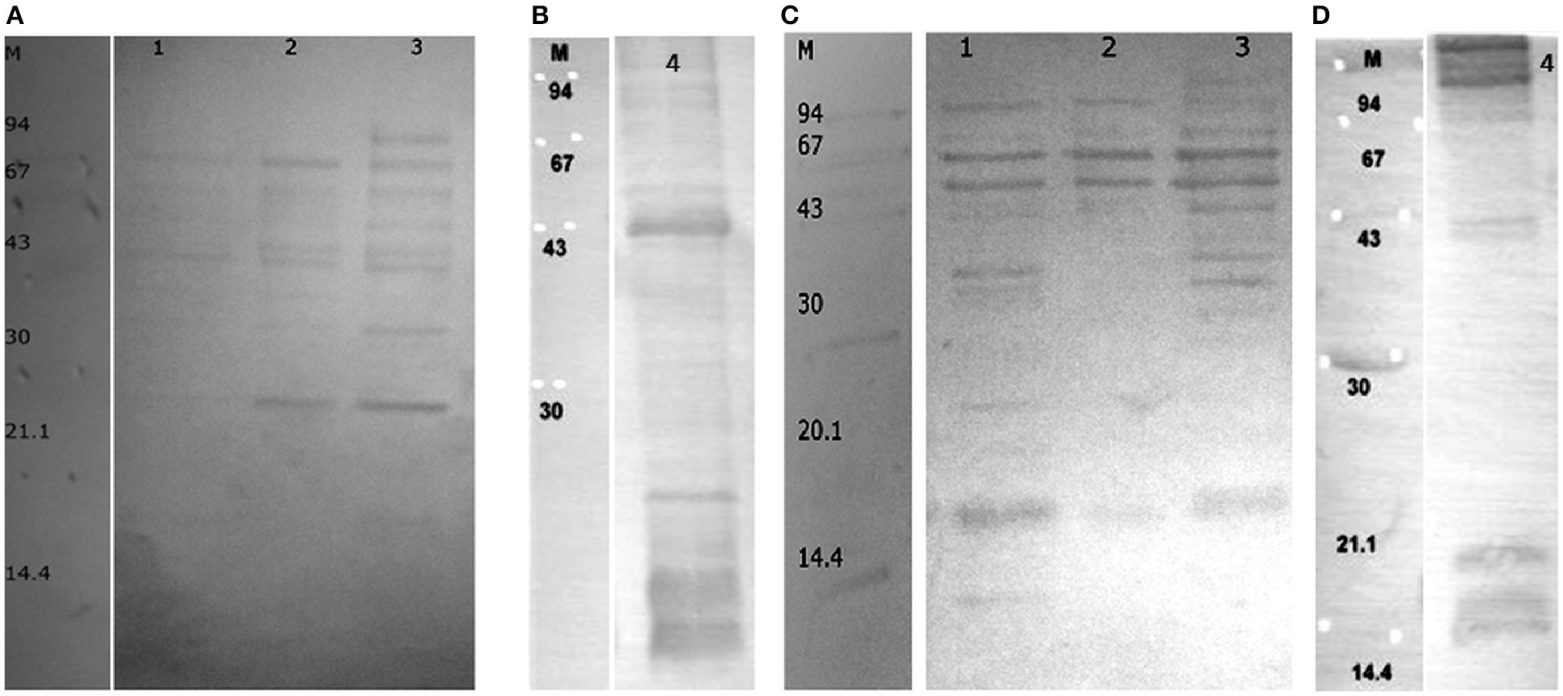
**Immunoblotting of proteins isolated from ***Bifidobacterium longum*** ssp. ***longum*** CCDM 372**. M, molecular mass marker [kDa]; line 1, protein extract of method III; line 2, protein extract of method IV; line 3, protein extract of method V; line 4, protein extracts of method VI; with sera against *Bifidobacterium longum* ssp. *longum* CCM 7952 **(A,B)** and *Bifidobacterium longum* ssp. *longum* CCDM 372 **(C,D)**. These images were taken from different blots, merged and the appropriate scaling was used.

Strain *B*. *longum* ssp. *longum* CCDM 372 produced several immunoreactive proteins, which reacted with homologous sera R-372, as well as with sera against strain CCM 7952, namely protein with molecular weight of about 94 kDa (Figure 4), 67 kDa (Figures 4A,C line, line 1, 2, 3), 55 kDa (Figure 4A line 3, Figure 4B line 1, 2, 3), 43 kDa (Figures 4A–D), 35 kDa (Figure 4A line 3, Figure 4C, line 1, 3), and a few between 14 and 25 kDa (Figures 4B–D).

**Figure 5:**
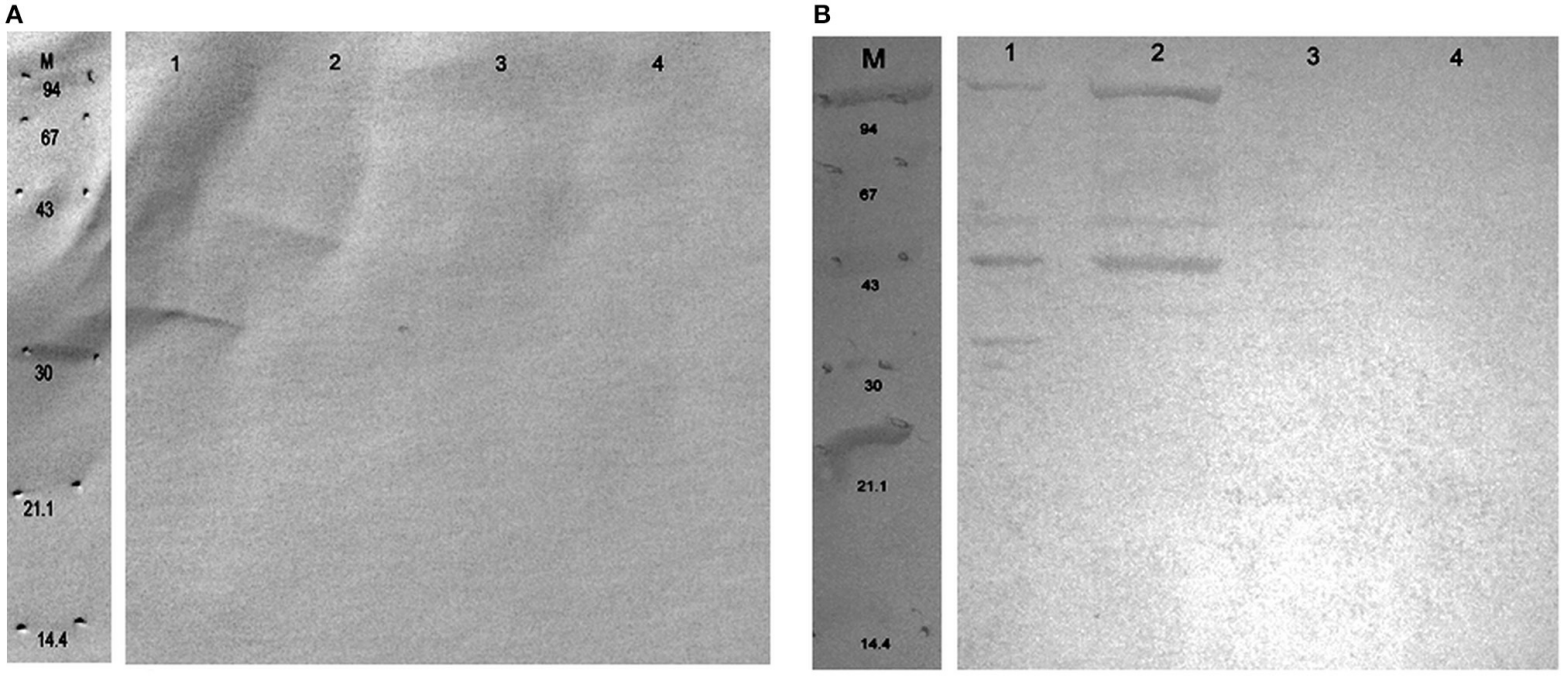
**Immunoblotting of proteins isolated from ***Bifidobacterium longum*** ssp. ***longum*** CCM 7952 (A) and CCDM 372 strain (B)**. M, molecular mass marker [kDa]; line 1, protein extract of method III; line 2, protein extract of method IV; line 3, protein extract of method V; line 4, protein extracts of method VI; with human sera obtained from patients with acute *Clostridium difficile*. These images were taken from different blots, merged, and the appropriate scaling was used.

**Figure 6:**
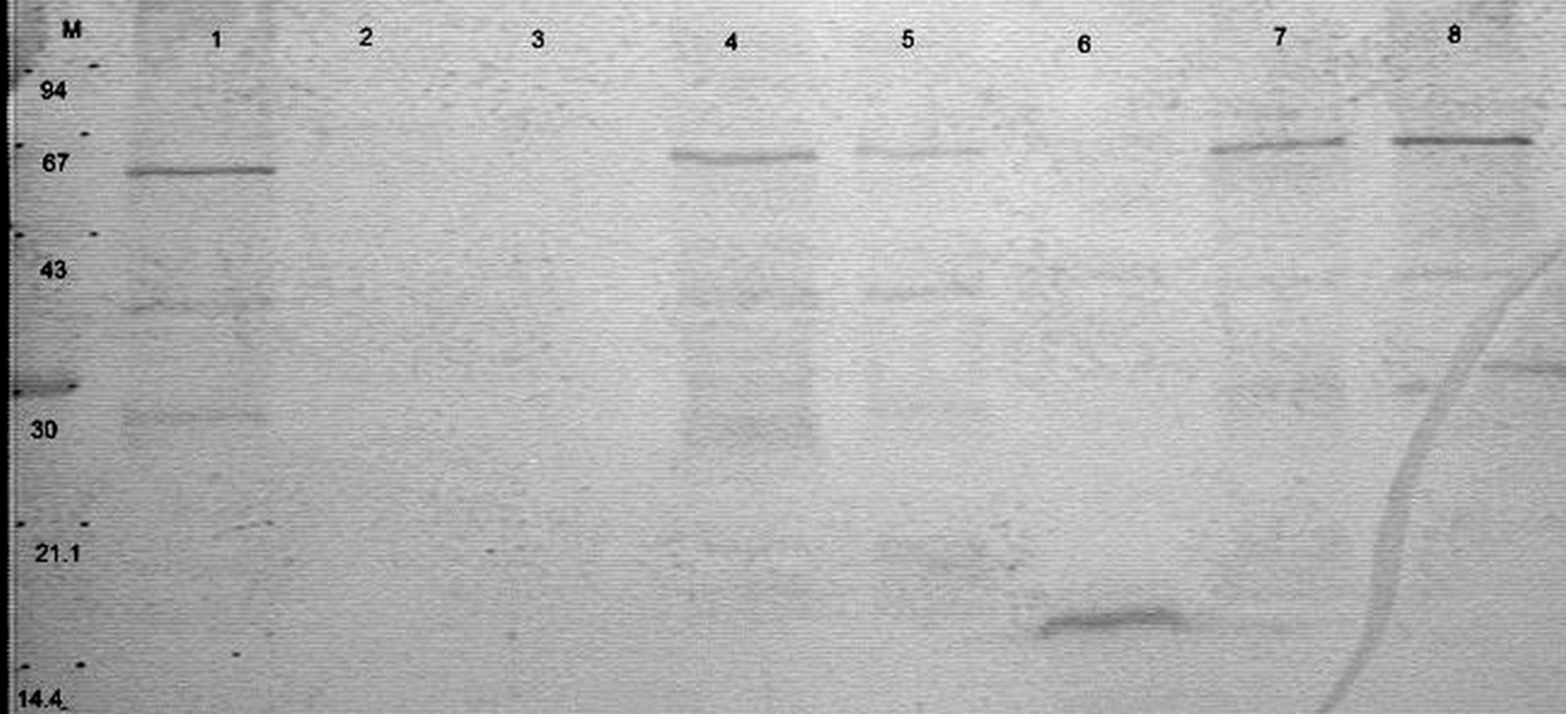
**Immunoblotting of proteins isolated from ***Bifidobacterium longum*** ssp. ***longum*** CCM 7952 (line 1–4) and CCDM 372 (5–8) strain**. M, molecular mass marker [kDa]; line 1, 5, protein extract of method III; line 2, 6, protein extract of method IV; line 3, 7, protein extract of method V; line 4, 8, protein extracts of method VI; with rabbit sera against *Lactobacillus paracasei* LOCK 0912.

Considering human sera, we didn't observed reactivity of protein extracts with healthy adult sera or cord blood sera, but we noticed a reactivity of proteins with molecular weight of about 94 and 45 kDa isolated from CCDM 372 strain with sera from patients with active *Clostridium difficille* infection (Figure 5). We also tested the cross-reactivity with rabbit sera against *Lactobacillus* species and we found a broad reactivity of protein isolated from both strains with molecular weight around 60 kDa (Figure 6).

**Figure 7:**
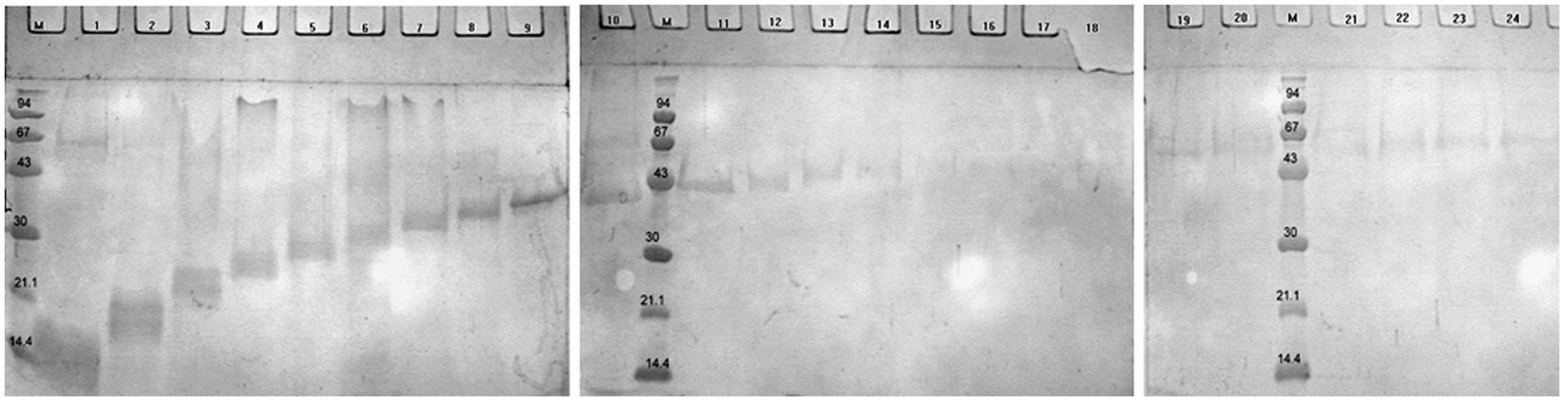
**The SDS-PAGE profile of a separated proteins isolated from ***Bifidobacterium longum*** ssp. ***longum*** CCDM 372 in the presence of M-molecular mass marker [kDa] by continuous-elution electrophoresis (Prep-Cell apparatus Model 491 Bio-Rad)**. Samples are electrophoresed through a cylindrical gel. As molecules migrate through the gel matrix, they separate into bands. Individual bands migrate off the bottom of the gel where they pass directly into the patented elution chamber for collection. The resulting liquid fractions (2 ml) were pooled (5 fractions), dried and analyzed on SDS-PAGE. Gels were stained with Coomassie Brilliant Blue.

**Figure 8:**
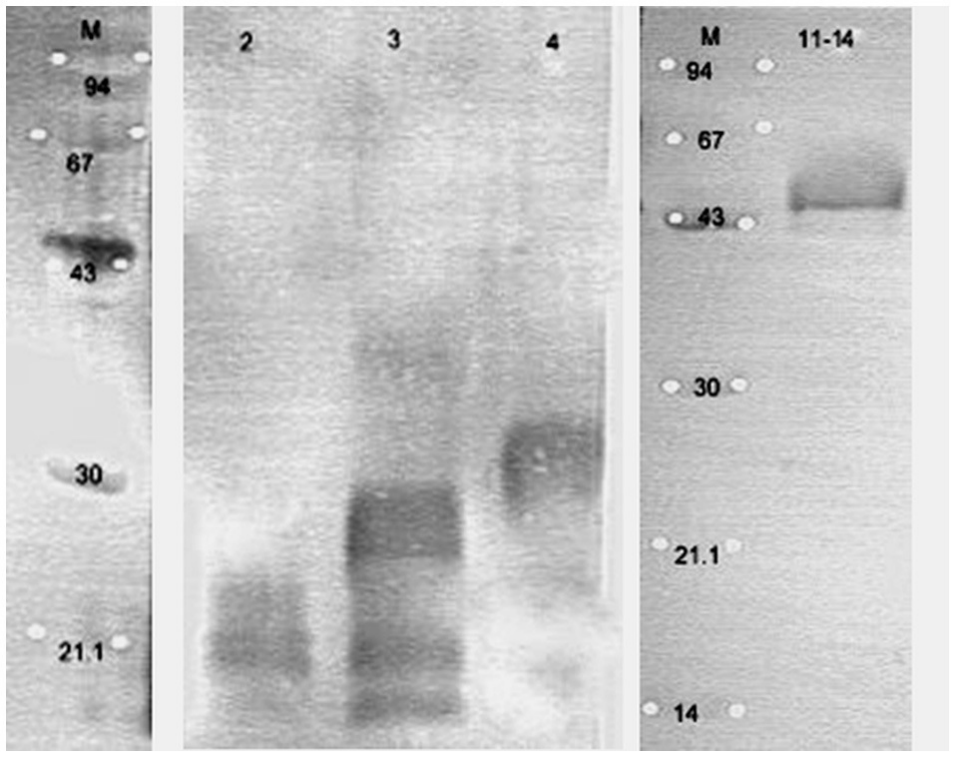
**The selected immunoblots of separated proteins isolated from ***Bifidobacterium longum*** ssp. ***longum*** CCDM 372 reacting with homologous serum**. Line 2, fraction 2; line 3, fraction 3; line 4, fraction 4; line 11–14, pooled fraction 11, 12, 13, and 14 obtained by continuous-elution electrophoresis (Figure 7). These images were taken from different blots, merged, and the appropriate scaling was used.

The protein extracts were purified using electrophoretic preparation using Prep-Cell apparatus and re-analyzed in Western blot. The representative results are shown on Figures 7, 8. Spots showing strong immunoreactivity features were cut from the gel and analyzed in LC-MS/MS. Proteins were identified by comparative analysis of peptides masses (NCBI, UniProt databases) using MASCOT and statistical analysis. Results of sequencing shown as most likely homologs of isolated and analyzed proteins are summarized in Table 2.

**Table 2:** **Identification of immunoreactive proteins isolated from ***Bifidobacterium*** strain**.

**Strain number**	**Estimated molecular weight of isolated protein [kDa]**	**Homologous protein name Accession No**.	**Nominal molecular weight of homologous proteins [kDa]**	**Origin of homologous protein**	**Homology [%]**	**Method of isolation**	**Immunoreactivity**
*Bifidobacterium longum* ssp. *longum CCM 7952*	43	Enolase ZP_00120494.2	46.6	*Bifidobacterium longum* DJO10A	38	VI	Rabbit sera against *B. longum* ssp. *longum* CCM 7952
	20	Aspartokinase WP_007051616.1	18.6	*Bifidobacterium longum* NCC2705	47	V	Rabbit sera against *B. longum* ssp. *longum* CCDM 372
	55	Pyruvate kinase ADC85999.1	52.5	*Bifidobacterium animalis* subsp. *lactis* BB12	70	IV, V	Rabbit sera against *B. longum* ssp. *longum* CCDM 372
	67	Molecular chaperone DnaK NP_695712.1	66.9	*Bifidobacterium longum* NCC2705	30	III, IV, V	Rabbit sera against *B. longum* ssp. *longum* CCDM 372
	60	Hypothetical protein BIFLAC_06461 ZP_02963269.1	62	*Bifidobacterium animalis* subsp. *lactis* HN019	30	III, VI	Rabbit sera against *L. paracasei* LCOK 0912
*Bifidobacterium longum* ssp. *longum CCDM 372*	40	Sugar ABC transporter ATP-binding protein NP_695858.1	40.7	*Bifidobacterium longum* NCC2705	37	VI	Sera from germ mice colonized with *Bifidobacterium longum* ssp. *longum* CCDM 372
	94	Not identified			III, IV, V, VI	Rabbit sera against *B*. *longum* ssp. *longum* CCDM 372
	66	Penicillin-binding protein 3 peptidoglycan synthetase EDT88982.1	65.3	*Bifidobacterium animalis* subsp. *lactis* HN019	65	III, IV, V	Rabbit sera against *B. longum* ssp. *longum CCM 7952 or* CCDM 372
	55	Pyruvate kinase WP_014697813.1	52.6	*Bifidobacterium animalis* subsp. *animalis* ATCC 25527	53	III, IV, V	Rabbit sera against *B. longum* ssp. *longum* CCDM 372
	43	Phosphoglycerate kinase NP_695890.1	46.6	*Bifidobacterium longum* NCC2705	39	III, IV, V, VI	Rabbit sera against *B. longum* ssp. *longum* CCM 7952 or CCDM 372
	35	Transaldolase ZP_04664439.1	39.7	*Bifidobacterium longum* subsp. *infantis* CCUG 52486	24	III, V	Rabbit sera against *B. longum* ssp. *longum* CCDM 372
	30	1-(5-phosphoribosyl)-5-[(5-phosphoribosylamino)methylideneamino] imidazole-4-carboxamide isomerase ZP_02963768.1	25.7	*Bifidobacterium animalis* subsp. *lactis* HN019	50	VI	Rabbit sera against *B. longum* ssp. *longum* CCM 7952 or CCDM 372
	22.6	50S ribosomal protein L5 ZP_02963325.1	22	*Bifidobacterium animalis* subsp. *lactis* HN019	40	VI	Rabbit sera against *B. longum* ssp. *longum* CCM 7952 or CCDM 372
	20	30S ribosomal protein S9 BAJ69893.1	17.5	*Bifidobacterium longum* subsp. *infantis* ATCC 15697	73	III	Rabbit sera against *B. longum* ssp. *longum* CCM 7952 or CCDM 372
	17	30S ribosomal protein S16 Q8G7G1.1	16.4	*Bifidobacterium longum* NCC 2705	79	III	Rabbit sera against *B. longum* ssp. *longum* CCM 7952 or CCDM 372
	14	50S ribosomal protein L15 WP_008783622.1	15.8	*Bifidobacterium longum* subsp. *infantis* ATCC 15697	60	III	Rabbit sera against *B. longum* ssp. *longum* CCM 7952 or CCDM 372
	45	Glyceraldehyde 3-phosphate dehydrogenase C KFI73119.1	39.3	*Bifidobacterium minimum*	40	III	Human sera from patients with acute *C. difficile* infection
	60	Hypothetical protein BIFLAC_06461 ZP_02963269.1	62	*Bifidobacterium animalis* subsp. *lactis* HN019	30	III, V, VI	Rabbit sera against *L*. *paracasei* LOCK 0912

The immunogenic spot with molecular weight around 43 kDa isolated from *B. longum* ssp. *longum* CCM 7952 reacted with homologous sera was identified as enolase (38% protein sequence coverage), while other protein cross-reacted with sera against *B. longum* ssp. *longum* CCDM 372 were identified as aspartokinase (47%), pyruvate kinase (70%), molecular chaperon DnaK (30%). It is noteworthy to highlight that immunoreactive spot with molecular weight around 40 kDa isolated from CCDM 372 strain reacted as the only one with sera from gnotobiotic mice colonized with this strain was identified as sugar ABC transporter ATP-binding protein (37% protein sequence coverage). We observed the highest protein sequence coverage of 66 and 55 kDa proteins isolated from *B. longum* ssp. *Longum* CCDM 372. This proteins have been identified as the penicillin-binding protein 3 peptidoglycan synthetase (65% protein sequence coverage) and pyruvate kinase (53% protein sequence coverage), respectively. The spots of 43 and 35 kDa of CCDM 372 were characterized by a low homology with phosphoglycerate kinase (39%) and transaldolase (24%), respectively. It has to be underline, that all above mentioned protein were isolated regardless of the chosen method. However, when we analyzed the protein with small molecular weight (less than 30 kDa) we observed a differences. Surprising, by method VI we were able to isolate from strain CCDM 372 two immunoreactive protein, namely 1-(5-phosphoribosyl)-5-[(5-phosphoribosylamino)methylideneamino] imidazole-4-carboxamide isomerase (50% protein sequence coverage) and 50S ribosomal protein L5 (40% protein sequence coverage), whereas by method III we isolated three completely different proteins: 30S ribosomal protein S9, 30S ribosomal protein S16 and 59S ribosomal protein L15 with 73, 79, and 60% protein sequence coverage, respectively. The immunoreactive protein with molecular weight about 45 kDa of CCDM 372 strain reacting with human sera from patients with acute *Clostridium difficile* infection was recognized as glyceraldehyde 3-phosphate (40%). Interestingly, spot with molecular mass of 60 kDa from CCDM 372 and CCM 7952 reacting with sera against *L*. *paracasei* LOCK 0912 was identified as hypothetical protein BIFLAC_06461 (30%).

## Discussion

The insight into the *Bifidobacterium*–host interaction mechanism, should be elucidated by identification the biological function of bacterial components like polysaccharide or proteins. It is believed that these antigens play a crucial role in the first line of the contact with host cells i.e., they are involved in the intestinal colonization by facilitating contact with epithelium cells and modulate the immune response. However, our knowledge about immunoreactive proteins of *Bifidobacterium* strains are poor. Recently, Talja et al. (2014) established the prevalence of serum IgG, IgM, and IgA antibodies specific for *Bifidobacterium adolescentis* and *Bifidobacterium longum* proteins in young children with or without type 1 diabetes, however without their detail characterization. In our studies we focused on identification of potentially immunoreactive/immunogenic proteins of two strains isolated from human origin: *Bifidobacterium longum* ssp. *longum* CCM 7952 and *Bifidobacterium longum* ssp. *longum* CCDM 372 using electrophoretic, immunoblotting and mass spectrometry method. The reactivity with different mouse, human or immunized rabbit sera was heterogeneous and varied between methods of isolation and serum samples. This could be explained by a distinct mechanism of each vaccination or host immune system. First, we noticed that the proteins isolated from strain *B. longum* ssp. *longum* CCDM 372 are recognized by different mouse, rabbit or human sera, whereas those from strain CCM 7952 reacted only with immunized rabbit sera. Second, strain CCDM 372 can induce the production of antibodies in mono-colonized gnotobiotic mice mouse compared to the strain CCM 7952. Moreover, the detected immunoreactive bands isolated from strain CCDM 372 show the cross-reactivity with antibodies against *Lactobacillus* species and sera of patients with *Clostridium difficile* infection. This observation may suggested, that the large amount of polysaccharide slime surrounding the bacterial cell of CCM 7952 strain is probably shielding other surface molecules from host immune cells and suppress antibody responses against them. This is in line with our previously studies of *Lactobacilli* proteins and polysaccharides (Fanning et al., 2012; Górska et al., 2014, 2016). Moreover, we showed that specific proteins isolated form tested strains are strains-specific i.e., enolase, whereas other like pyruvate kinase share a cross-reactivity.

Regarding the immunoreactive protein identified in this paper, most of them are well-known as highly conserved cytoplasmic or ribosomal proteins. However, they could be very often exposed on the bacterial surface to take an additional activities, e.g., they could be responsible for health benefit to the host, can be involved in adhesion to epithelium cells or interaction with plasminogen (Wang et al., 2013). The proteins, which have been found to serve two or more functions are described as moonlighting proteins. Mostly, the studies on moonlighting proteins concern the pathogenic bacteria like *Streptococcus pyogenes, Mycoplasma pneumoniae*, enteropathogenic *Escherichia coli, Staphylococcus aureus* and fungi or parasites (Henderson, 2014; Karkowska-Kuleta and Kozik, 2014) and recently, research has focused on the roles of moonlighting proteins in probiotics (Lebeer et al., 2010).

The immunogenic protein of strain CCM 7952 was identified as enolase. Candela et al. (2009) demonstrated that four bifidobacterial species, a health-promoting member of the human intestinal microbiota: *B*. *longum, B*. *bifidum, B*. *breve*, and *B*. *lactis* share an enolase on a cell surface and plasminogen-binding activity. Recently, Wei et al. (2014), identified that enolase and elongation factor Tu serve as surface receptors for *B. longum* NCC2705 binding to human plasminogen which could inhibit the adhesion of *B. longum* NCC2705 to Caco-2 cells and suggested that these enzymes are involved in the protective role played by *B. longum* NCC2705 in defense against enteric pathogens. Interestingly, the surface enolase may not only play a role as a receptor for human plasminogen, but also as a fibronectin-binding protein (Castaldo et al., 2009).

Apart from immunogenic enolase, the main immunoreactive proteins of *B. longum* ssp. *longum* CCM 7952 were identified as aspartokinase, pyruvate kinase and molecular chaperon DnaK. Chaperon DnaK from *B*. *animalis* subsp. *lactis* BI07 has been visualized on the bacterial cell surface and the recombinant DnaK protein showed a high affinity for human plasminogen (Candela et al., 2010).

Our most striking observation was that, colonization of germ-free mouse with *B*. *longum* ssp. *longum* CCDM 372 elicited antibodies against 40 kDa protein recognized as sugar ABC transporter ATP-binding protein. To induce the beneficial effects on the host, bifidobacteria must be able to survive and persist in the gut. This is possible not only by using an adhesion or colonization factors, but also by production specific substances allowed them to recruitment the energy from the fermentable carbohydrates not absorbed or metabolized by the host. He et al. (2007) demonstrated that bifidobacteria are able to utilize a wide range of catabolic pathways using enzymes for metabolism of lactose, glucose and galactose. Schell et al. (2002) provided a genome analysis of *Bifidobacterium longum* and identified several major proteins, including 26 solute binding proteins of ABC transporter systems which could be important in terms of colonization in the gut and nutrient availability or involved in the immunomodulatory activity of bifidobacteria. It was also shown that the ATP binding protein of ABC transporter for sugars increased abundance during interaction of *B*. *longum* with Caco-2 (Wei et al., 2014) and they are expressed under oxygen stress (Xiao et al., 2011) or under bile exposure (Ruiz et al., 2009).

One of the key findings of this work is the identification of several immunogenic protein of CCDM 372 reacting with rabbit homologs sera and sera against strain CCM 7952. In particular, peptidoglycan synthetase penicillin-binding protein 3, pyruvate kinase, phosphoglycerate kinase (PGK), transaldolase and different ribosomal proteins. Ruiz et al. (2009) found five ribosomal proteins of *Bifidobacterium longum* biotype *longum* NCIMB 8809 to be overproduced in the presence of bile. Previously, several surface-associated ribosomal proteins have been also identified in *Streptococcus suis* (Aranda et al., 2009), *Streptococcus pyogenes* (Ventura et al., 2003) and *Lactobacillus rhamnosus* GG (Sánchez et al., 2009) where they are involved in sensing the environmental changes. Transaldolase has been already shown to be release into medium (Sánchez et al., 2008). In addition, it was also detected in the extracellular proteome of *B. animalis* subsp. *lactis* BB12 (Gilad et al., 2011). Recently, González-Rodríguez et al. (2012) indicated that this protein could act as an important colonization factor favoring *Bifidobacteria* establishment in the gut.

Interestingly, we indicated that non-immune human sera, not only of adult blood donors, but also from umbilical cord sera, didn't contain antibodies recognizing proteins isolated from both bifidobacteria strains. However, we observed the cross-reactivity of 45 kDa protein isolated from *B*. *longum* subsp. *longum* CCDM 372 with sera from patients with *Clostridium difficile* infection. This protein was identified as glyceraldehyde 3-phosphate dehydrogenase (GAPDH), which is one of the earliest examined moonlighting protein and their multifunctional nature as adhesins as well as plasminogen receptors had been well characterized (Jin et al., 2005; Terrasse et al., 2012; Giménez et al., 2014). The immune response against GAPDH have been described for numerous pathogens i.e., *S. aureus* (Kerro-Dego et al., 2006), *Edwarsiella tarda* (Li et al., 2012), *Streptococcus iniae* (Ra et al., 2009), *Streptococcus agalactiae* (Liu et al., 2013). In our previous work we indicated that GAPDH is at least one of immunogenic protein of *Lactobacillus johnsonii* 142 and immunoreactive protein of probiotic *L. rhamnosus* LOCK 0900 and suggested the importance of this protein in cross-talk between bacteria and their host (Górska et al., 2016). Recently, it has been suggested that GAPDH possesses role in host-immune responses, modification of intracellular signaling and evasion from immune surveillance of the host (Perez-Casal and Potter, 2016). The ability of GAPDH to bind to extracellular matrices, modulation of host-immune responses, a role in virulence and surface location has prompted investigators to postulate that GAPDH may be a good vaccine candidate for protection against numerous pathogens. However, it is reasonable to think that immune response against the GAPDH may also result in deleterious effect to the host due to cross-immune reactions. Alignment of the *Bifidobacterium longum* GAPDH protein sequence with some pathogens i.e., *Clostridium difficile, Shigella flexneri, Salmonella enterica, Escherichia coli* and probiotic bacteria i.e., *L. rhamnosus* GG, *Lactobacillus reuteri* JCM 1112, *Lactobacillus helveticus* CNRZ32 or human GAPDH proteins using Clustal Omega reveals a high degree of homology. For instance, the homology of GAPDH between *B. longum* and human, *C. difficile, E.coli* is around 42, 50, and 60%, respectively and between *B. longum* and *L. rhamnosus* GG shared homology of 68%. It will be interesting to identify epitopes of these protein that evoke protective immune response and open the possibility to use *Bifidobacterium* GAPDH as an antigens for development of vaccines.

This study demonstrates for the first time that glycolytic enzymes, other metabolic enzymes, molecular chaperones or ribosomal protein from human *Bifidobacterium* isolates are able to induce the immune response and elicited antibodies in strain dependent manner. This observation raise the question of the impact of this protein in the mechanism of the *Bifidobacterium*–host interaction. We indicated that the protein extraction methods and analysis of immunoreactivity could be suitable to differentiate among the species of bifidobacteria and their biological function. The immunoreactive proteins identified in our study open a new potential possibilities of using them as medically important molecules i.e., vaccines development; however the further analysis are needed to provide the details about the biological nature of specific antigens and to select the best candidates.

## Author contributions

SG designed, coordinated and conceived of the study, performed the protein identification and drafted the manuscript, ED carried out the immunoassays, A Rudawska, A Razim carried out protein isolation and purification, EB prepared the anti-rabbit and human sera, MS prepared the bifidobacterium strains, DS prepared the mouse sera, HK contributed reagents/materials and help to draft the manuscript, AG was a supervisor and helped to draft the manuscript. All authors read and approved the final manuscript.

## Funding

This work was supported by grants CZ.3.22/2.1.00/09.01574 and CZ.3.22/2.1.00/13.03892. co-funded by the European Regional Development Fund under Operational Programme Cross-border Cooperation Czech Republic–Republic of Poland 2007–2013, under the European Territorial Cooperation Objective and by grant MOBILITY 7AMB16PL006 of the Ministry of Education, Youth and Sports of the Czech Republic Publication supported by Wroclaw Centre of Biotechnology, programme The Leading National Research Centre (KNOW) for years 2014–2018.

### Conflict of interest statement

The authors declare that the research was conducted in the absence of any commercial or financial relationships that could be construed as a potential conflict of interest. The reviewer VD and handling Editor declared their shared affiliation, and the handling Editor states that the process nevertheless met the standards of a fair and objective review.
